# The Temporal Dynamics of the Labeling Algorithm During Natural Language Comprehension: Neural Evidence for Phrase Grammatical Type Generation

**DOI:** 10.1162/NOL.a.264

**Published:** 2026-07-01

**Authors:** Zhenghui Sun, Feipeng Chen, Shaodong Gui, Yajiao Shi, Yiming Yang

**Affiliations:** School of Linguistic Sciences and Arts, Jiangsu Normal University, Xuzhou, China; College of Biomedical Engineering and Instrument Sciences, Zhejiang University, Hangzhou, China; Suzhou Xingze Experimental School, Suzhou, China; College of Humanities and Communication, Zhejiang University of Finance & Economics, Hangzhou, China; Linguistic Science Laboratory of Jiangsu Normal University, Laboratory of Philosophy and Social Sciences at Universities in Jiangsu Province, Xuzhou, China; Collaborative Innovation Center for Language Ability, Jiangsu Normal University, Xuzhou, China

**Keywords:** EEG, grammatical type, labeling algorithm, representational similarity analysis (RSA), syntax-semantics interface

## Abstract

Language, as a uniquely human faculty, combines words into hierarchical phrases with diverse syntactic-semantic relations (e.g., subject–predicate and modifier–head phrases). These phrase types are theoretically generated through the labeling algorithm at the syntax-semantics interface. Nevertheless, previous research has focused predominantly on the syntactic system, whereas the neural basis and temporal dynamics of labeling remain poorly understood. To address this gap, we developed the Head-Anchored Labeling Manipulation approach. This method manipulates labeling by embedding the same Mandarin noun–verb dual-category words as heads in tightly controlled modifier–head constructions, where the head alone determines the phrase’s grammatical type. The representational similarity analysis of EEG data revealed that labeling representations emerged during both the early and middle stages: 192–227, 290–318, 330–360, and 385–416 ms following the dual-category word. Labeling emerged as early as ~200 ms, concurrent with syntactic Merge, and continued into the N400 window. These results demonstrate that labeling dynamically links syntax to conceptual–intentional system and generates phrase types by determining the constituent head. Its early engagement challenges syntax-first models and supports parallel interactive accounts. Moreover, combinability representations (520–596 and 604–632 ms), together with a late event-related brain potential negativity (420–700 ms) elicited by the dual-category word in the baseline condition, reflect increased difficulty in reconciling semantic associations with atypical syntactic configurations. Together, these findings provide clear electrophysiological evidence for the temporal dynamics of labeling and elucidate the processes at the syntax-semantics interface.

## INTRODUCTION

Language, as a uniquely human cognitive faculty, is fundamentally characterized by the ability to construct hierarchical structures. This ability enables discrete lexical items to be combined into phrases that encode diverse syntactic-semantic relations, such as subject–predicate, predicate–object, and modifier–head phrases. Within the Minimalist Program (Chomsky, 2013, 2023), these phrase types are proposed to be derived by a critical operation at the syntax-semantics interface: the labeling algorithm. By assigning categorical labels, the labeling algorithm yields asymmetric hierarchical structures and thereby plays an indispensable role in syntactic structure building (Goucha et al., 2017). Nevertheless, its neural underpinnings remain largely unexplored, particularly the temporal dynamics of the labeling algorithm during online language processing.

Theoretically, the labeling algorithm operates at the interface between the syntactic rule system and the conceptual-intentional system (Berwick et al., 2013; Chomsky, 2013). Within the syntactic system, Merge first combines two elements into a symmetric set without inherent headedness or category. Subsequently, at the syntax-semantics interface, the labeling algorithm applies to this set and assigns a grammatical type. This operation determines its headedness and converts the symmetric configuration into an asymmetric phrase (Berwick et al., 2013; Chomsky, 2013, 2023; Chomsky et al., 2019, 2023; Dehaene et al., 2015). For example, in the phrase *the ship*, labeling identifies the determiner *the* as the head, resulting in a determiner phrase with a clear hierarchical organization. This process shows that Merge builds structures while labeling identifies them, playing an essential role in establishing interpretable syntactic and semantic relations. Therefore, within the Minimalist framework which posits that labeling operates at the syntax–semantics interface, its neural implementation is expected to emerge temporally at the interface between early syntactic structure building and semantic interpretation.

Despite its theoretical centrality, empirical research on labeling remains scarce. From the perspective of Marr’s levels of analysis, Minimalist syntax primarily operates at the computational level, characterizing the abstract computations of the language faculty. However, it does not specify the algorithmic processes of real-time parsing or their implementation in neural tissue. Bridging this gap requires neurocomputational models that translate formal grammatical operations into testable neural hypotheses. Recent frameworks, such as the ROSE model (Representation, Operation, Structure, Encoding; Murphy, 2015, 2024, 2025), aim to provide precisely this bridge by proposing explicit oscillatory dynamics for core syntactic operations, including labeling. Previous neurolinguistic studies on hierarchical structure building have primarily focused on the neural mechanisms of Merge (Ding et al., 2016; Fedorenko et al., 2016, 2020; Fitch & Hauser, 2004; Friederici, 2017; Hagoort, 2019; Hauser et al., 2002; Murphy et al., 2022, 2024; Nelson et al., 2017; Pallier et al., 2011). In contrast, the neural dynamics of labeling at the syntax-semantics interface have received considerably less attention. Although direct investigations of labeling are still limited, related research can be broadly categorized into three strands, with a third, emerging one beginning to directly examine labeling.

First, studies have examined the neural dissociation between lexical categories, especially nouns versus verbs, across semantic, grammatical, and phonological domains (Bi et al., 2007; Caramazza & Hillis, 1991; Liu et al., 2008; Yang et al., 2002; Yu et al., 2011, 2012; for reviews, see Liang & Han, 2010; Vigliocco et al., 2011). Notably, Liu et al. (2008) employed Chinese dual-category words (兼类词/*jiān lèi cí*, also referred to as category-flexible words, i.e., lexical items that can function grammatically as both nouns and verbs) to dissociate the neural correlates of nouns and verbs in contexts. However, such studies do not provide direct evidence for labeling per se, as the observed effects cannot be specifically attributed to labeling over other co-occurring processes. To specifically isolate the labeling mechanism, the combinatorial context must be held constant, with grammatical type serving as the only variable. All other factors—including semantics, phonology, orthography, word order, syntactic structure, and head position—must be rigorously controlled.

Second, studies using category-violation paradigms have shown that lexical category violations can elicit the Early Left Anterior Negativity (ELAN, 200–300 ms) (Friederici et al., 1993; Neville et al., 1991; Osterhout & Holcomb, 1992), a component interpreted as a neural signature of early, automatic syntactic processing (Friederici, 1995, 2017). However, the ELAN paradigm suffers from several critical limitations: (1) the conditions being compared differ not only in grammatical category but also in syntactic well-formedness and semantic accessibility; (2) violation-based designs may confound multiple cognitive processes (Kaan & Swaab, 2002, 2003) or even introduce experimental artifacts (Steinhauer & Drury, 2012); and (3) ELAN effects lack robust cross-linguistic generalizability (Steinhauer & Drury, 2012; van den Brink & Hagoort, 2004). Consequently, the ELAN is more likely to reflect a composite of multiple early linguistic processes and is therefore unlikely to serve as a specific neural marker of labeling.

Third, most recent studies have begun to focus on labeling in linguistic composition. For instance, studies using artificial grammar paradigms have examined how category-based labeling facilitates the acquisition of complex syntactic rules in second language learning (Chen et al., 2018, 2019). Other work has identified neural correlates associated with different phrasal types (Schell et al., 2022; Zhang et al., 2022). Notably, Zhang et al. (2022) highlighted the role of the left angular gyrus in representing and distinguishing fine-grained types of phrasal grammatical relations. Extending this line of research, Zhang et al. (2024a) further pinpointed the left posterior middle temporal gyrus as a key region involved in differentiating verb–noun category relations.

Nevertheless, the temporal dynamics of labeling remain unresolved. To date, no study has directly determined when labeling occurs during real-time language processing, leaving it unclear whether it emerges in early or middle stages. This issue is crucial because the timing of labeling governs the processing of phrasal grammatical types and constitutes a pivotal step in constructing coherent hierarchical structures. Given that labeling operates at the syntax-semantics interface, previous research offers partial insights into its mechanisms, but the exact temporal locus remains unknown. Semantic integration is reliably indexed by the N400 (300–500 ms) (Kutas & Federmeier, 2011; Kutas & Hillyard, 1980; Lau et al., 2008). For syntax, early components such as ELAN, the Early Syntactic Negativity (Hasting & Kotz, 2008), and the syntactic Mismatch Negativity (Hasting et al., 2007) have been reported. More recently, Sun et al. (2025) showed that hierarchical structure building via Merge begins around 200 ms, from which point syntax and semantics exhibit a dynamic, parallel-interactive mode of processing. This result supports a dynamic parallel-interaction model of syntax–semantics processing (Lyu et al., 2019; Murphy et al., 2022, 2024; Parrish & Pylkkänen, 2022). These findings suggest that labeling may plausibly emerge in an early (approximately 200 ms) window with a maximum delay of less than 400 ms.

The key methodological challenge, then, is how labeling can be directly investigated within natural language composition. An ideal approach would contrast labeled versus unlabeled syntactic structures. However, in natural language, all well-formed phrases are inherently labeled. Encouragingly, Mandarin provides a unique testing ground: its dual-category words are categorically ambiguous between noun and verb categories in the absence of syntactic context (Liu et al., 2008). For example, the word 工作 (*gōng zuò*, “work”) can function as a noun (as in 一份工作/*yī fèn gōng zuò*, “a work”) or a verb (as in 拼命地工作/*pīn mìng de gōngzuò*, “to work hard”). This property inherently triggers different grammatical categories, providing a promising opportunity to investigate labeling while controlling all other variables. Furthermore, we embedded dual-category words into uniform modifier–head structures (e.g., adjective–noun vs. adverb–verb: 快乐的工作/*kuàilèdē gōngzuò* “a happy work” vs. 快乐地工作/*kuàilèdē gōngzuò* “work happily”). In modifier–head constructions, the head determines the syntactic category and interpretive properties of the entire phrase. This property provides a tractable structural framework for examining the effects of labeling. In short, we refer to this approach as the Head-Anchored Labeling Manipulation (HALM) approach.

Therefore, the present study developed the HALM approach to investigate the neural correlates and temporal dynamics of the labeling algorithm by using high-temporal-resolution EEG. The HALM approach isolates syntactic labeling by embedding dual-category words as heads in uniform modifier–head frames. In this paradigm, the head’s category (noun vs. verb) is the only variable differing across conditions by strictly controlling confounding variables at both the word and phrase levels (details in Methods, Experimental Design and Materials). In order to establish a condition without labeling, the Baseline (BL) condition without syntactic context was also included. Methodologically, we employed representational similarity analysis (RSA; Kriegeskorte et al., 2008) to examine the similarity of multidimensional neural activity patterns and theoretical models. This approach overcomes key limitations of traditional event-related brain potential (ERP) analysis, which relies on one-dimensional waveform averages across channels and cannot capture the representational content of neural activity. Guided by the theory of the syntax-semantics interface and recent evidence for early, dynamic parallel syntactic–semantic processing (Lyu et al., 2019; Murphy et al., 2022, 2024; Parrish & Pylkkänen, 2022; Sun et al., 2025), we explicitly predict that labeling primarily emerges during early stages of processing (around ∼200 ms) and unfolds dynamically thereafter.

## MATERIALS AND METHODS

### Participants

Thirty-eight university students (19 female, 19 male; aged 18–27 years) participated in the experiment. Two male participants were excluded from ERP analyses due to having fewer than 75% valid trials after artifact rejection (Luck, 2014), leaving 36 data sets for ERP analysis (mean age = 20.28 years, *SD* = 1.72). For the RSA, one additional female participant was excluded because all trials corresponding to one item were lost following artifact rejection, resulting in 35 data sets for RSA (mean age = 20.26 years, *SD* = 1.74). All participants were native Mandarin Chinese speakers, right-handed as assessed by the Edinburgh Handedness Inventory, and had normal or corrected-to-normal vision with no history of neurological or language disorders. To ensure experimental validity, participants were pre-screened to confirm that they understood the functional distinction between the particles 的 (*dē*) and 地 (*dē*) and reported no confusion regarding their usage in daily writing. Written informed consent was obtained from all participants, who received monetary compensation for participation. The study was approved by Jiangsu Normal University’s Academic Ethics Committee.

### Experimental Design and Materials

In the experiment, dual-category words were embedded within a uniform modifier–head phrasal structure. Within such constructions, the head determines the syntactic category and interpretive profile of the entire phrase. This design ensured maximal overlap between the two phrases in lexical content, phonological form, and basic semantic interpretation, as well as in syntactic configuration and head position. The only systematic difference lay in grammatical category. Accordingly, our manipulation targeted the grammatical status of the dual-category word in head position (i.e., the second word). Analyses therefore focused on the neural responses elicited by this critical word.

**Figure 1.**
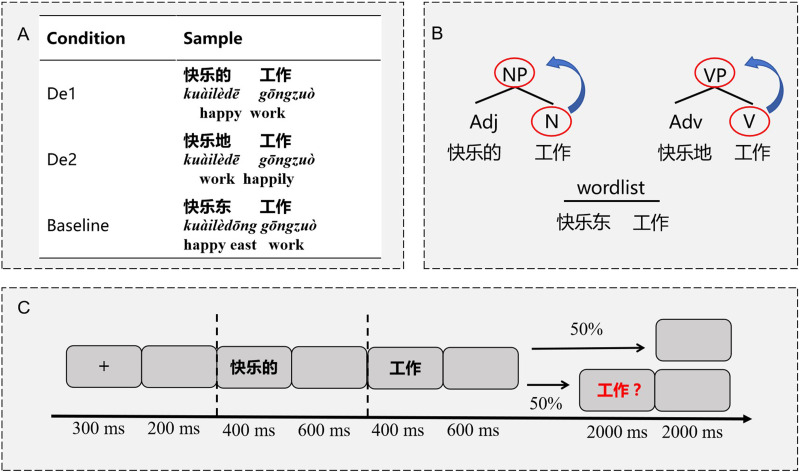
Experimental design and procedure. (A) Design and example of stimuli. (B) Schematic illustration of the design. Sequences with syntactic Merge form modifier–head hierarchical structures. In these constructions, the head (here, the dual-category word) determines the syntactic category and interpretive profile of the entire phrase; thus, the dual-category head serves as the anchor of the phrase type, with its category (noun or verb) specifying the phrase label. By contrast, sequences without syntactic Merge form flat, linear wordlists, serving as the baseline condition. In this case, the critical word (e.g., 工作, “work”) cannot undergo syntactic combination, leaving the dual-category word unlabeled and preventing the sequence from being assigned a phrase label. (C) The procedure of a trial. A one-word probe task was employed, appearing in 50% of all trials and distributed pseudo-randomly across all conditions and fillers. Each trial began with a 300 ms fixation cross (“+”), followed by a 200 ms blank screen. The two words were then presented sequentially, each for 400 ms, with a 600 ms blank interval between them. A probe word in red font (followed by a question mark) was then presented pseudo-randomly. Participants had 2,000 ms to decide whether the probe word had appeared in the current trial by pressing “1” for “yes” or “3” for “no.” No response was required when no probe word was presented. The inter-trial interval was 2,000 ms. De1: modifier–head construction in which an adjective + 的 (dē) precedes a dual-category word labeled as a noun head; De2: modifier–head construction in which an adverb + 地 (dē) precedes a dual-category word labeled as a verb head; NP: noun phrase; Adj: adjective; N: noun; VP: verb phrase; Adv: adverb; V: verb.

The experiment adopted a within-item design comprising 60 critical stimulus sets. Each set contained three conditions (Figure 1A–B), with each condition consisting of two words presented sequentially. In the De1 condition (adjective + 的 + dual-category word), the sequence formed an adjective-noun phrase (e.g., 快乐的—工作, “happy work”). In the De2 condition (adverb + 地 + dual-category word), it formed an adverb-verb phrase (e.g., 快乐地—工作, “work happily”). The adjectives and adverbs used in De1 and De2 were strictly matched items that share an identical surface form and root meaning, minimizing extraneous linguistic variability. In the BL condition, the functional morpheme in the first word was replaced with a content word, specifically a locative noun (e.g., 东/*dōng*, “east”; 西/*xī*, “west”; 南/*nán*, “south”; 北/*běi*, “north”). This substitution prevented the sequence from forming a syntactic structure, leaving the grammatical category of the dual-category word indeterminate and, consequently, the phrasal category of the entire string undefined. Thus, the baseline condition amounted merely to a word string linked by semantic association (e.g., 快乐东—工作, “happy east work”).

To ensure the quality of materials, we implemented a multi-stage selection procedure at both the lexical and phrasal levels. This rigorous process ensured that the dual-category words ultimately embedded in the modifier–head structure were free from pre-existing category biases, semantic disparities, and differences in phrasal familiarity or combinability. At the word level, noun–verb dual-category words were selected based on balanced categorical preferences and minimal semantic difference between their verbal and nominal uses. First, all candidate words were verified as genuine noun–verb dual-category words against both the *Dictionary of dual-category word discrimination* (Chen, 1990) and the *Modern Chinese Dictionary* (6th ed.; Institute of Linguistics, Chinese Academy of Social Sciences, 2012). This is the initial candidate word pool, comprising a total of 166 words. Words belonging to other categories (e.g., tri-category words such as 创新/ *chuàngxīn, meaning* “innovation/innovate/innovative”) were further excluded. Second, 38 undergraduates (none of whom participated in the main experiment) were recruited to rate the initial set of words on a 7-point scale (1 = *definitely a noun*, 7 = *definitely a verb*; 4 = *equally likely to be a noun or a verb*). This rating assessed whether these candidate words exhibited any categorical bias in everyday language use. Thirty-five valid questionnaires were retained, with validity determined by correct responses to ten obvious error words interspersed throughout the questionnaire. Words with mean ratings between 3.50 and 4.50 were selected. Subsequently, we excluded words with substantial meaning shifts between categories. The final word set (e.g., 工作—“work”) consisted of words with identical meanings in both grammatical categories. This step resulted in 125 candidate items.

At the phrase level, we ensured that both De1 and De2 phrases were commonly used and matched for familiarity and combinability. First, all modifier–head phrases containing dual-category words that met the above criteria were selected from the highest-frequency entries in the BCC Corpus (https://bcc.blcu.edu.cn/). Low-frequency phrases, even when semantically transparent, were excluded. Second, a separate group of 95 undergraduates was recruited to rate the familiarity and combinability of the phrases on a 5-point scale (89 valid data sets were retained for analysis). In the final set of 60 stimulus pairs, no significant differences were observed between the De1 and De2 conditions in terms of familiarity (*M* ± *SD*: 4.459 ± 0.318 vs. 4.433 ± 0.368; *t*(59) = 0.899, *p* = 0.371) or combinability (4.400 ± 0.311 vs. 4.391 ± 0.273; *t*(59) = 0.280, *p* = 0.781).

The 60 item sets were divided into three lists via a Latin square design, with each list containing 20 phrases per condition. Each list was presented in both forward and reverse orders, resulting in six counterbalanced versions. To improve the signal-to-noise ratio of the ERP responses and minimize data loss after artifact rejection, each condition was repeated three times per participant.

To prevent participants from developing a fixed expectancy strategy, especially given that all experimental materials were modifier–head phrases, an equal number of filler trials was added for each condition. Fillers for the De1condition followed an “adjective + adjective” pattern (e.g., 准确的—准确的/zhǔnquè *dē*—zhǔnquè *dē*, “accurate—accurate”), those for De2 followed an “adverb + adverb” pattern (e.g., 严格地—严格地/yángé *dē*—yángé *dē*, “strictly—strictly”), and fillers for BL followed an “XX东 + XX东” pattern (e.g., 勇敢东—勇敢东/*yǒnggǎn dōng*—yǒnggǎn dōng, “brave east—brave east”).

### Procedure

The experiment was conducted in a sound-attenuated, moderately lit, and electromagnetically shielded EEG laboratory. Participants were seated approximately 80 cm from the computer screen, with their eye level aligned to the center of the screen. They were instructed to minimize head and body movements, as well as to avoid unnecessary eye blinks, in order to reduce potential EEG artifacts. Prior to the formal EEG recording, they reviewed the task instructions, verbally confirmed their understanding, and completed a practice session.

The experimental task was programmed using E-Prime 3.0 (Psychology Software Tools, USA). All stimuli were presented in black font against a silver-white background. A one-word probe task was embedded in 50% of the trials and distributed pseudo-randomly across all conditions and fillers. Each trial (Figure 1C) began with a fixation cross (“+”) presented for 300 ms, followed by a 200 ms blank screen. The two words were then presented sequentially for 400 ms each, separated by a 600 ms blank interval. Subsequently, a probe word was presented in red font followed by a question mark for a duration of 2,000 ms. Participants were instructed to indicate whether the probe had appeared in the current trial by pressing “1” for “yes” or “3” for “no”. Response accuracy and reaction times were recorded. If no probe was presented, no response was required. The probe word could be the first word (25%), the second word (25%), or an unpresented but category-matched word (50%). The inter-trial interval was set at 2,000 ms. The response key assignment for left and right hands was counterbalanced across participants.

This implicit probe task was designed to (1) maintain participants’ attention throughout EEG recording and (2) avoid confounds from explicit plausibility judgments (e.g., conflict monitoring; Kaan & Swaab, 2002, 2003). The use of single-word probes combined with a moderate stimulus onset asynchrony (SOA) preserved the temporal independence of word-level processing while allowing natural linguistic interactions between words.

To mitigate the limitations associated with Event-related design (e.g., potential confounds between conditions) and Block design (e.g., susceptibility to anticipatory strategies), we implemented a mini-block design that incorporates the advantages of both approaches. Each mini-block comprised five experimental trials and five matched filler trials, presented in a pseudorandomized sequence. To minimize the risk of forced syntactic combination in the BL condition resulting from exposure to the experimental conditions, participants first completed a block composed of BL trials, followed by a block containing the experimental conditions (De1 and De2). The presentation order of De1 and De2 trials was counterbalanced across participants.

Following the EEG session, participants completed a post-experiment questionnaire based on the experimental items presented during the recording (fillers excluded). For each item, participants were asked to rate the following on a 5-point Likert scale: (1) the combinability of the two words, ranging from *very low* to *very high*; and (2) the perceived grammatical category of the second word in its context, where 1 represented 100% noun, 5 represented 100% verb, and 3 represented equally likely.

### EEG Data Acquisition and Preprocessing

Continuous EEG signals were recorded using a 64-channel Ag/AgCl electrode cap (Neuroscan, USA), with electrodes placed according to the standard international 10–20 system (Jasper, 1958). Vertical electrooculography was recorded using electrodes placed above and below the left eye, and horizontal electrooculography was recorded using electrodes placed at the outer canthi of both eyes. The left mastoid electrode was used as the online reference, and all electrode impedances were kept below 5 kΩ. The EEG signals were recorded at a sampling rate of 1000 Hz with a band-pass filter of 0.10 Hz to 100 Hz.

Data preprocessing was performed in MATLAB with the EEGLAB (Delorme & Makeig, 2004) and ERPLAB (Lopez-Calderon & Luck, 2014) toolboxes. The signals were offline re-referenced to the average of the left and right mastoids and filtered with a 0.10–30 Hz bandpass filter. The data were downsampled to 500 Hz, and artifacts from ocular, muscle, and electrocardiographic sources were removed using independent component analysis (ICA). Epochs were extracted from −1,200 to 1,000 ms relative to the critical word onset, covering the first word (−1,000 to 0 ms) and the critical word (0 to 1,000 ms). Baseline correction was performed using the interval from −1,200 to −1,000 ms relative to the critical word onset (corresponding to −200 to 0 ms relative to the first word onset). Trials with signals exceeding ±90 *μ*V were excluded. The trial retention rates were comparable across conditions (De1: 94.5%, De2: 94.5%, BL: 94.6%).

### Data Analysis

#### Representational similarity analysis of spatiotemporal neural patterns and behavioral measures

**Figure 2.**
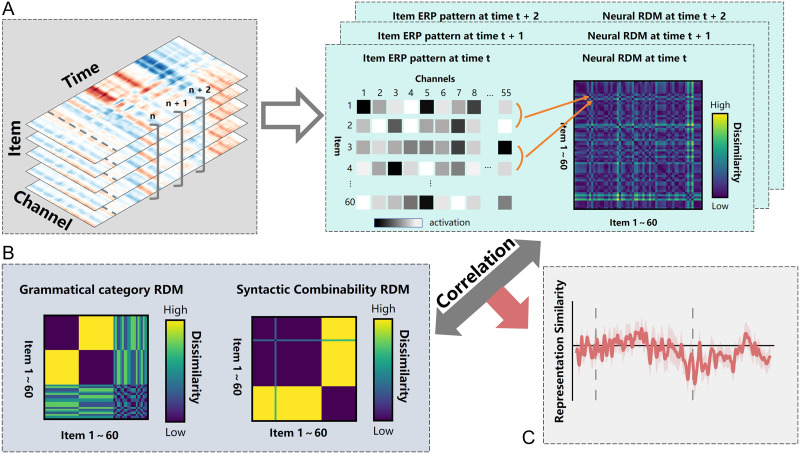
Item-based representational similarity analysis (RSA) pipeline. (A) For each participant and time point, EEG epochs were averaged across three repetitions of each item to obtain item-level event-related potentials. Multivariate activation vectors based on all 55 electrodes were extracted to construct neural RDMs (item × item) over time. (B) Behavioral RDMs were derived from post-experiment ratings of grammatical category (left) and combinability (right), representing dissimilarity between each pair of items. (C) At each time point, neural RDMs were correlated with behavioral RDMs using Spearman’s rank correlation, generating time-resolved neural-behavioral similarity profiles. These similarity values were then averaged across participants to produce grand-average traces that indicate the time windows during which scalp-wide neural activity patterns encoded grammatical-category and combinability information. ERP = event-related brain potential; RDM = Representational Dissimilarity Matrix.

We performed an item-based spatial RSA using the NeuroRA package (Lu & Ku, 2020) in Python to assess the similarity of scalp-wide neural activity patterns over time. This approach allowed us to identify significant time windows during which similarity effects emerged. The analysis consisted of three main steps. First, for each participant, we constructed an item-based neural Representational Dissimilarity Matrix (RDM) at every time point (see Figure 2A). Specifically, since each item was presented three times, we averaged the EEG epochs corresponding to each item to obtain item-level average waveforms first. The data were epoched to cover both the first word (−200 to 1,000 ms) and the second word (−200 to 800 ms). For each participant, item, and time point, we then computed a neural activity pattern vector comprising signals from all 55 electrodes, representing the multivariate EEG response for each item at each specific time point. We then computed neural RDMs at each time point by calculating 1 − Pearson correlation across these multichannel activation patterns, which provided a time-resolved measure of pairwise dissimilarity between items. Second, for each participant, we constructed behavioral RDMs based on post-experiment ratings: one for grammatical category ratings and another for combinability ratings. Specifically, behavioral RDMs were computed using the absolute differences in 1–5 Likert-scale ratings between items, serving as an index of perceived dissimilarity (representative sample RDMs for one participant are illustrated in Figure 2B). Third, for each participant and time point, we quantified the similarity between the neural RDM and each behavioral RDM using Spearman’s rank correlation. This procedure generated time-resolved similarity profiles for both the grammatical category and combinability. To visualize the group-level time course, the similarity values were averaged across participants at each time point, producing grand-average similarity traces for each dimension (see Figure 2C).

To evaluate the statistical significance of the neural–behavioral similarity, we conducted cluster-based permutation tests (Maris & Oostenveld, 2007) over the entire epoch with 2,000 permutations.

#### ERP statistical analysis using linear mixed-effects models

To complement the RSA, ERP analyses were also performed. Based on both visual inspection and prior studies (Sun et al., 2025), one window was analyzed: 420–700 ms following the onset of the second word, corresponding to the late time window reported in Sun et al. (2025). Additionally, we analyzed a 300–500 ms window following the onset of the first word (i.e., −700 to −500 ms relative to the second word onset). Mean amplitudes were calculated within each time window. For the second word, the average amplitude during the −200 to 0 ms pre-stimulus interval was included as a covariate to account for potential baseline shifts.

Following prior ERP studies (Shi et al., 2022; Zhu et al., 2018), electrodes were grouped into four regions of interest (see Supplementary Figure S1; Supporting Information can be found at https://doi.org/10.1162/NOL.a.264): left anterior (F1, F3, F5, FC1, FC3, FC5), right anterior (F2, F4, F6, FC2, FC4, FC6), left posterior (CP1, CP3, CP5, P1, P3, P5), and right posterior (CP2, CP4, CP6, P2, P4, P6).

We performed linear mixed-effects models (LMM) analyses to compare mean amplitude differences across conditions within each time window, using the lme4 (1.1-27.1; Bates et al., 2015) and lmerTest (3.1-3; Kuznetsova et al., 2017) packages in R version 4.1.1. In each model, the across-time average amplitude of each electrode, trial, and participant was treated as an individual observation of the dependent variable for each time window. Fixed effects included the following predefined comparisons: De1 vs. Baseline, De2 vs. Baseline, and De1 vs. De2. Additionally, fixed effects for electrode regions included two hemisphere comparisons (left hemisphere vs. right hemisphere) and two region comparisons (anterior region vs. posterior region). Interactions among all these fixed effects were also included in the model. The random effect structure was determined through an iterative, stepwise model selection procedure using the Akaike Information Criterion (AIC; Akaike, 1974) and likelihood ratio tests with chi-squared distribution (Matuschek et al., 2017). Starting with a full model that incorporated random intercepts for participants, trials, and electrodes as well as all related random slopes (Barr et al., 2013), random slopes were removed step by step. At each step, simplified models were compared using AIC values. The model with the smallest AIC was selected as a candidate and then compared to the more complex model from the previous step via a likelihood ratio test. A *p* value < 0.2 (Matuschek et al., 2017) indicated that the more complex model should be retained. This iterative process continued until an optimal model was identified or all random slopes were removed. Models that failed to converge were excluded from the analysis. For significant interaction effects, simple effects analyses were conducted using separate LMMs, following the same model selection procedure as described above (Chang et al., 2020).

In the Results, we report parameter estimates (*b*), standard errors (*SE*), *t*-statistics, and *p* values for significant fixed effects related to experimental conditions at the critical word.

## RESULTS

Behavioral data indicated high engagement for each participant. Accuracy rates exceeded 97% (*M* = 99.04%, *SD* = 1.06%), and reaction times ranged from 553 to 1,049 ms (*M* = 726.65 ms, *SD* = 129.41 ms).

### RSA Results

This study employed the same experimental parameters as Sun et al. (2025), including the paradigm, task, and SOA, in investigating phrase structure. Therefore, we followed their approach and divided the processing stream into three approximate stages: early (200–300 ms), middle (300–420 ms), and late (420–700 ms). It is important to note that this division is intended as a purely descriptive approximation of the temporal evolution of neural representations. We acknowledge that ERP/RSA timing alone cannot establish computational equivalence. Accordingly, we do not interpret the observed time windows as reflecting multiple independent stages.

### Grammatical Category Representation

**Figure 3.**
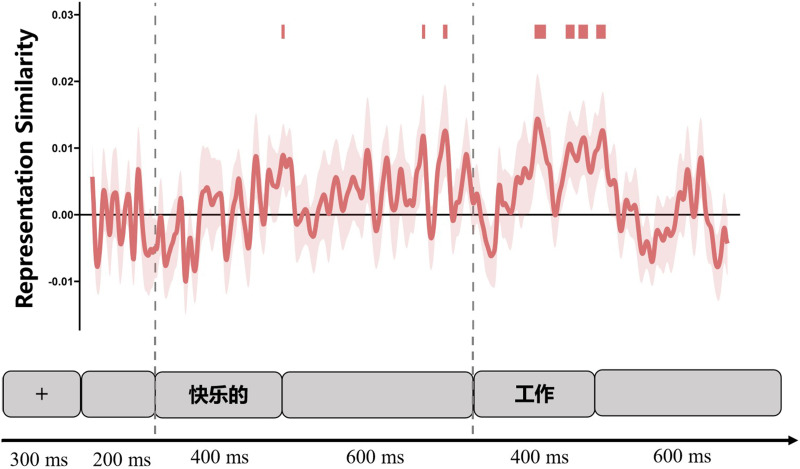
Representational similarity results of grammatical category. The upper panel displays the representational similarity values, with the top red line indicating statistically significant time windows. The lower panel shows corresponding examples of trials. Significant similarity effects during the presentation of the second (critical) word were observed within four time windows: 192–227, 290–318, 330–360, and 385–416 ms (cluster-based permutation test, *p*s < .05). Additionally, significant similarity effects during the presentation of the first word were observed in three time windows: 395–406, 838–848, and 904–918 ms (cluster-based permutation test, *p*s < .05). Shaded areas represent ±1 standard error of the mean (*SEM*) across participants.

Grand average similarity waveforms over time are displayed in Figure 3 for grammatical category representation. Statistical analyses revealed that during the presentation of the critical word, significant representational similarity effects associated with grammatical category emerged within four distinct time windows: 192–227, 290–318, 330–360, and 385–416 ms (cluster-based permutation test, *p*s < .05). These results indicate that the brain encodes grammatical category information during both early and middle stages of critical word processing.

During the presentation of the first word, statistical analyses revealed that significant representational similarity effects associated with grammatical category were also observed at 395–406, 838–848, and 904–918 ms (cluster-based permutation test, *p*s < .05). These results indicate that grammatical category information was mainly encoded during middle-stage processing of the first word and just before the onset of the second word.

### Combinability Representation

**Figure 4.**
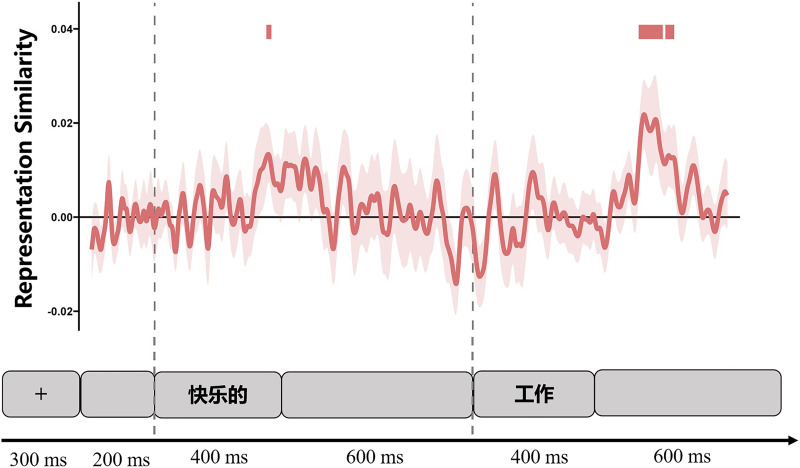
Representational similarity results of combinability. The upper panel displays representational similarity values, with the top red line indicating statistically significant time windows. The lower panel shows corresponding examples of trials. Significant similarity effects during the presentation of the critical word were observed during the late stage, specifically within 520–596 ms and 604–632 ms (cluster-based permutation test, *p*s < .05). No significant combinability representations were observed in the early or middle stages. Additionally, significant similarity effects during the presentation of the first word were observed at 350–366 ms (cluster-based permutation test, *p*s < .05). Shaded areas represent ±1 *SEM* across participants.

Grand average similarity waveforms over time are displayed in Figure 4 for combinability representation. Statistical analyses revealed that during the presentation of the critical word, significant representational similarity effects associated with combinability were found at 520–596 ms and 604–632 ms during the late processing stage (cluster-based permutation test, *p*s < .05). No significant combinability representations were detected during either the early or middle stages. During the presentation of the first word, statistical analyses revealed that significant combinability representations were observed at 350–366 ms (cluster-based permutation test, *p*s < 0.05).

### LMM Results

**Figure 5.**
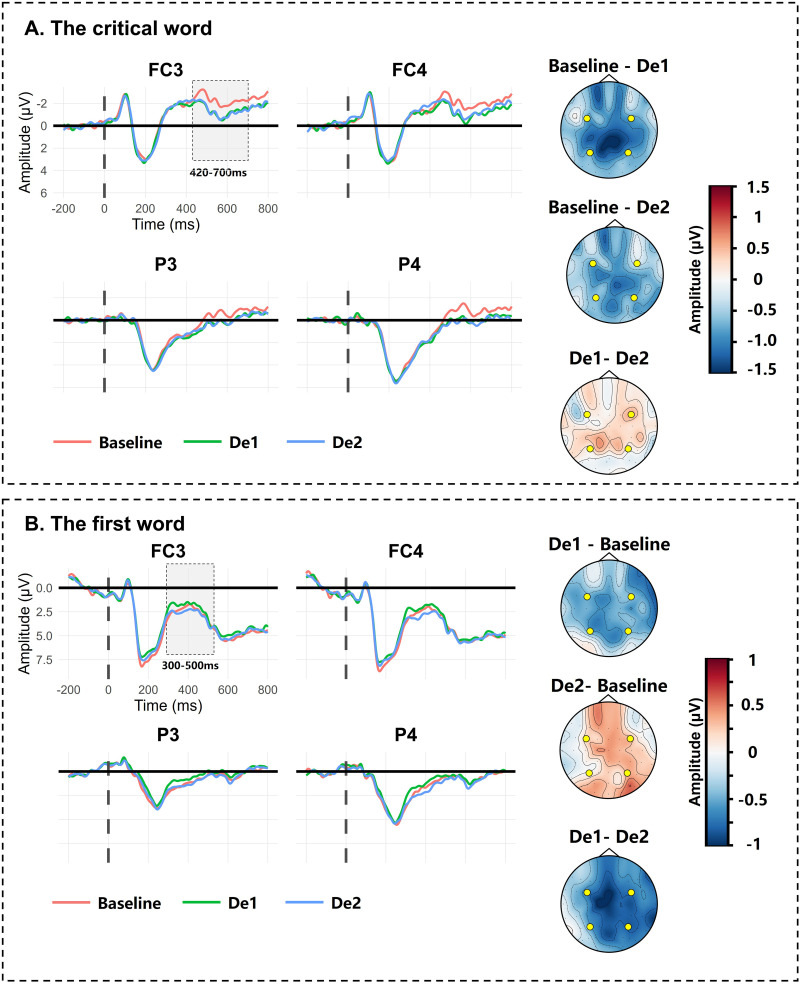
Grand average ERP waveforms at representative electrodes. (A) During the presentation of the critical word, the Baseline (BL) condition elicited greater late negativity than both De1 (adjective–noun) and De2 (adverb–verb) conditions in the 420–700 ms time window. No significant difference was found between De1 and De2. Topographical maps on the right illustrate the scalp distributions of differences among the three conditions. (B) During the presentation of the first word, the De1 condition elicited larger negativity than both BL and De2 conditions in the 300–500 ms time window; the BL condition also elicited larger negativity than the De2 condition in this time window. Topographical maps on the right show the scalp distributions of differences among the three conditions. Gray bars at electrode FC3 mark the analyzed time windows. Channels FC3 and FC4 correspond to left and right anterior regions, respectively; P3 and P4 represent left and right posterior regions. The four yellow circles in the topographical maps mark the four representative electrodes. Time is displayed relative to word onset in milliseconds (ms); amplitude is shown in microvolts (*μ*V). De1: adjective + 的 (dē) + dual-category head word, forming an adjective–noun phrase; De2: adverb + 地 (dē) + dual-category head word, forming an adverb–verb phrase.

Grand average ERP waveforms over time are displayed in Figure 5A during the presentation of the critical word. Within the 420–700 ms time window, the BL condition elicited significantly larger late negativity than both the De1 condition (*b* = −0.425, *SE* = 0.023, *t* = 18.129, *p* < 0.001) and the De2 condition (*b* = −0.380, *SE* = 0.023, *t* = 16.218, *p* < 0.001) . A significant Condition × Region interaction was observed for the De2 vs. BL contrast (*b* = −0.103, *SE* = 0.047, *t* = −2.205, *p* = 0.027). Simple effects analysis showed that the BL condition elicited stronger negativity than the De2 in both anterior (*b* = −0.760, *SE* = 0.211, *t* = 3.602, *p* < 0.001) and posterior regions (*b* = −0.964, *SE* = 0.197, *t* = 4.886, *p* < 0.001). This late negativity effect converges with RSA results that revealed combinability representations within the same time window.

Grand average ERP waveforms over time are displayed in Figure 5B during the presentation of the first word. Within the 300–500 ms time window, the De1 condition elicited significantly larger negativity than the BL condition (*b* = −0.235, *SE* = 0.023, *t* = −10.402, *p* < 0.001) . The BL condition, in turn, elicited larger negativity than the De2 condition (*b* = 0.072, *SE* = 0.023, *t* = 3.196, *p* = 0.001). Furthermore, the De1 condition elicited significantly larger negativity than the De2 condition (*b* = −0.617, *SE* = 0.045, *t* = −13.792, *p* < 0.001). This effect occurred within a time window consistent with the N400 component reported in previous studies (Kutas & Federmeier, 2011) and converges with RSA results that revealed grammatical category representation and combinability representation in the same window.

## DISCUSSION

The present study developed the HALM approach to examine the neural correlates and temporal dynamics of the labeling algorithm using high-temporal-resolution EEG. This approach places noun–verb dual-category words in the head position of tightly controlled modifier–head constructions, making the head the sole anchor of the phrase while holding all other factors constant. This design ensures that the grammatical category (noun vs. verb) of the dual category word exclusively dictates the label of the entire phrase, offering a direct and rigorously controlled investigation into labeling processes. The spatiotemporal RSA revealed several significant time windows for labeling effects following the critical word onset: 192–227 ms, 290–318 ms, 330–360 ms, and 385–416 ms. These results indicate that the human brain begins to encode grammatical category relations and execute the labeling algorithm as early as ∼200 ms, with these processes continuing dynamically through subsequent time windows. Converging with recent evidence that syntactic Merge occurs around 200 ms (Sun et al., 2025), our results suggest that labeling operates in parallel with Merge during early processing. This concurrent engagement of Merge within the syntactic system and labeling at the syntax–semantics interface demonstrates immediate interactions between syntax and semantics. These interactions give rise to asymmetric hierarchical structures enriched with syntactic–semantic relations. This finding poses a direct challenge to syntax-first models and offers robust support for interactive accounts of language processing. At later stages (520–596 ms, 604–632 ms), RSA revealed combinatory representations, while ERP showed a late negativity (420–700 ms) in the baseline condition. These converging results suggest that deep syntactic–semantic integration occurs during this period.

This study presents clear scalp EEG evidence for the labeling algorithm in natural phrase comprehension. Previous studies have investigated related processes using artificial grammar learning (Chen et al., 2018, 2019) or comparisons across different phrase structures (Schell et al., 2022; Zhang et al., 2022, 2024a). However, these approaches did not yield direct evidence of the labeling algorithm itself. To isolate the algorithm and capture such evidence, our paradigm was designed around one essential requirement: the phrases must differ exclusively in their grammatical category as assigned by labeling while being rigorously matched on all other linguistic dimensions, including semantics, phonology, and syntactic constructions. Building on earlier work using dual-category words (Liu et al., 2008), we selected Mandarin noun–verb dual-category words (e.g., 工作, “work”). These words share identical forms and highly similar meanings across categorical uses, and we excluded words with significant semantic shifts. By embedding the same dual-category word as the head of modifier–head structures (e.g., 快乐的工作 “happy work” vs. 快乐地工作 “work happily”), we ensured that only its noun/verb status determined the phrase label, with all other lexical and phrasal variables held constant. Methodologically, unlike approaches that rely on waveform amplitudes averaged across channels, RSA captures multidimensional neural activity patterns from all channels and directly tests the similarity between theoretical models and neural representations. Capitalizing on these advantages, we precisely identified the temporal window in which the neural representation of the labeling algorithm emerges. Our results uncover the neural basis of labeling and offer clear empirical validation of the labeling hypothesis in syntactic theory (Chomsky, 2013, 2023).

Beyond demonstrating the operation of the algorithm itself, this study also provides clear electrophysiological evidence for the temporal dynamics of grammatical category labeling. Whereas previous research has begun to uncover the spatial neural representations of grammatical categories (Zhang et al., 2022, 2024a), the temporal aspects of these processes have remained largely unexplored. Here, the spatiotemporal RSA results reveal that the brain begins to encode grammatical category information through labeling as early as ∼200 ms. This timeline converges well with recent EEG findings indicating that syntactic Merge emerges within the 200–280 ms time window in minimal hierarchical structures (Sun et al., 2025). This suggests that although labeling and Merge are theoretically attributed to different systems, they operate simultaneously during real-time processing. In other words, the brain engages semantic resources during syntactic combination, leading to an immediate syntax-semantics interaction in which grammatical role assignments are implemented. Furthermore, labeling processes persist into the middle stage of processing, aligning with the widely reported N400 component, which reflects deeper syntax-semantics integration (Kutas & Federmeier, 2011; Kutas & Hillyard, 1980; Lau et al., 2008). The identification of multiple distinct time windows indicates that labeling is not a unitary event but a dynamic process that unfolds over time.

This work bridges theoretical linguistics and cognitive neuroscience, demonstrating that labeling is not merely an abstract grammatical construct, but a neurocognitive process with a distinct temporal signature (Coopmans & Zaccarella, 2023; Matchin et al., 2024; Murphy, 2015, 2024, 2025; Poeppel & Embick, 2005). In particular, our results provide empirical constraints for emerging neurocomputational models that seek to ground syntactic operations in specific neural dynamics. For example, the ROSE model (Murphy, 2024, 2025) formalizes syntactic structure building within a framework of multi-band neural oscillations. The very early onset (∼200 ms) of category-sensitive effects observed here falls within the temporal range associated with the engagement of low-frequency (δ/θ) processes during initial syntactic composition in this framework. Furthermore, the sustained neural differentiation we observed may be compatible with the dynamic, cross-frequency interactions proposed by such models. To our knowledge, recent work has examined the oscillatory dynamics underlying structural inference during category processing (Yang et al., 2025). Importantly, while the present study did not directly measure oscillatory activity, our findings provide precise temporal parameters that can serve as empirical anchors for future time–frequency investigations testing the specific predictions of the ROSE model and related neurocomputational approaches.

More importantly, these findings directly elucidate the core generative mechanism of the labeling algorithm in producing distinct phrase grammatical types by demonstrating its role in dynamically linking syntactic computation with the conceptual–intentional system. Our results support a processing model in which labeling and Merge are not sequential steps but function in an integrated manner. Syntactic rules provide a hierarchical framework that serves as a scaffold for conceptual content. Within this scaffold, syntax and semantics interact in real time to assign grammatical roles and determine the constituent head, thereby transforming the symmetric output of Merge into an asymmetric hierarchical phrase. This integrated mechanism—whereby syntax and semantics jointly determine categorical roles and the constituent head—is what theoretical linguistics identifies as labeling (Berwick et al., 2013; Chomsky, 2013, 2023; Chomsky et al., 2019, 2023). Through this process, distinct syntactic-semantic relations are configured immediately, giving rise to different phrase grammatical types. Ultimately, labeling captures the continuous and dynamic interplay between syntactic computation and conceptual structure, binding formal grammatical rules to meaning in real time. Through this process, hierarchical linguistic structures mirror the architecture of human cognition (Chomsky et al., 2023; Dehaene et al., 2015; Ding, 2025; Fan et al., 2025; Gui et al., 2020; Hauser et al., 2002).

Critically, the early engagement of labeling (∼200 ms) challenges traditional syntax-first models (Frazier & Fodor, 1978; Friederici, 2011, 2017) and provides clear empirical support for interactive accounts of syntax-semantics integration (Bastiaansen & Hagoort, 2006; Fromont et al., 2020; Hagoort, 2003, 2013, 2019; MacDonald et al., 1994; Marslen-Wilson & Tyler, 1980; Wang et al., 2013, 2015; Yang et al., 2021; Zhang et al., 2013). Specifically, syntax-first serial accounts posit an initial, autonomous stage of syntactic analysis prior to semantic integration. The latter is typically associated with the N400 component (300–500 ms; Kutas & Federmeier, 2011; Kutas & Hillyard, 1980; Lau et al., 2008). In contrast, our results demonstrate that syntactic and semantic processes interact from the earliest stages of processing. This finding, while based on Chinese materials, converges with recent evidence from multiple languages and methodological approaches (Lyu et al., 2019; Murphy et al., 2022, 2024; Parrish & Pylkkänen, 2022; Sun et al., 2025). It indicates that core syntactic and semantic processes, including labeling, begin much earlier than traditionally posited. This finding supports a dynamic and interactive view of language processing that generalizes across typologically diverse languages. The early syntax-semantics interaction also explains the difficulties previous studies (e.g., Pylkkänen, 2019) faced in disentangling them. While linguistic theories often assume a degree of independence between syntax and semantics (Chomsky, 2023), recent evidence from real-time processing suggests that these processes interact dynamically during online comprehension (e.g., Lyu et al., 2019; Murphy et al., 2022, 2024; Parrish & Pylkkänen, 2022; Sun et al., 2025). This, in turn, highlights the value of our minimally contrasting design and sensitive RSA approach in capturing these rapid and intertwined processes.

The present study achieves ecological validity while avoiding the limitations of violation-based paradigms. Although ELAN studies (Friederici, 1995; Friederici et al., 1993; Neville et al., 1991; Osterhout & Holcomb, 1992) have reported early negativity in response to category violations within time windows similar to those observed here, violation paradigms inherently introduce syntactic anomalies, semantic disruption, and interface breakdowns (Kaan & Swaab, 2002, 2003; Steinhauer & Drury, 2012). These factors lead to neural responses that reflect a mixture of multiple cognitive processes. In contrast, our approach employed the processing of natural phrases combined with multivariate RSA to directly detect representational differences across labeling conditions. This approach provides more specific evidence, offers superior ecological validity, and allows for greater interpretive precision.

During later stages of language comprehension, deeper integration of syntactic and semantic processing is likely to occur. The RSA results revealed combinability representations primarily in the 520–596 ms and 604–632 ms windows, converging with the late negativity elicited in the BL condition between 420–700 ms in the ERP analysis. Notably, the absence of such representations in the earlier stages may be attributed to our experimental design. Unlike fully meaningless strings, the baseline condition in this study replaced functional morphemes with locative nouns (e.g., 快乐东—工作, “happy east—work”), allowing participants to utilize residual semantic cues (e.g., the relation between “快乐”, “happy” and “工作”, “work”) to automatically initiate syntactic Merge. This finding is consistent with previous evidence that semantic context facilitates syntactic processing (Sun et al., 2025; Zhang et al., 2024b). Furthermore, participants reported that they actively ignored anomalous elements (e.g., 东 “east”) and attempted forced integration, suggesting that early syntactic processing is highly automatic (Batterink & Neville, 2013). In contrast, a sustained late negativity was observed in the BL condition between 420 and 700 ms, accompanied by robust representations of phrasal combinability in the RSA analysis within the same time window. The presence of combinability-related neural representations suggests that participants attempted phrasal integration despite disrupted syntactic structure in the BL condition. The late negativity is therefore compatible with increased cognitive effort during integration, potentially reflecting difficulty in reconciling semantic associations with atypical syntactic configurations (Friederici, 2017; Sun et al., 2025).

However, we acknowledge that such sustained negativities may also index more general processing demands, including heightened semantic integration load, anomaly detection, or failure to construct a coherent representation (Jiang et al., 2009; Jiang et al., 2013). In addition, it should be further noted that the absence of the P600 component in the present study may be attributed to the low demands of the implicit task on complex syntactic analysis, given that the P600 is typically elicited by syntactic violations or syntactic-semantic reanalysis in sentence-level context (Bornkessel-Schlesewsky & Schlesewsky, 2008; Brouwer et al., 2017; Fritz & Baggio, 2020). Consistent with this finding, Neufeld et al. (2016) also reported no P600 when investigating phrase structure building using a picture-word matching task, even with pseudowords.

Prior to the critical word, category-specific neural responses were observed between 300 and 500 ms during the processing of the first word, with adjectives eliciting stronger N400 effects than adverbs. RSA further revealed representations of both grammatical category (395–406 ms) and combinability (350–366 ms) during the processing of the first word. These findings suggest that the functional morpheme preceding the critical word provided distinct grammatical category relations. This interpretation is consistent with Liao et al. (2020), who reported a stronger N400 component for the Chinese particle “的”(*dē*, attributive) compared to “地” (*dē*, adverbial) and “得” (*dē*, complement) when used incorrectly, indicating distinct neural processing mechanisms for “的” during integration. However, N400 amplitude is sensitive to a range of factors, including conceptual–semantic access and lexical–distributional properties (Kutas & Federmeier, 2011). Therefore, the observed N400 differences may also reflect intrinsic distinctions between adjective and adverb uses of the same lexical root, such as morphological complexity or semantic specificity.

Furthermore, our RSA results revealed categorical representations emerging immediately before the onset of the critical head noun or verb (838–848 ms and 904–918 ms). This suggests that the functional morphemes de1 (的) and de2 (地) serve as potent predictive cues that pre-activate the grammatical category of the upcoming word (Matchin, 2023). This finding is consistent with previous accounts that emphasize context-based cues in rapid predictive processing (Brothers et al., 2023; Kutas & Federmeier, 2011; Pan et al., 2024; Wang et al., 2018). Our experimental design—featuring an implicit task, a long SOA, and a balanced trial structure—was intended to minimize strategic expectancy effects driven by task requirements or superficial stimulus patterns. Therefore, the finding highlights the intrinsic predictive role of these grammatical markers. Importantly, we interpret such prediction not as an alternative to labeling, but as a facilitating mechanism that interacts with early category-based structural integration, within which labeling is instantiated. Finally, we acknowledge that future work should further disentangle how predictive mechanisms and compositional operations jointly contribute to early phrase-structure building (Murphy, 2025). In addition, it should be noted that the present study employed an implicit task to minimize interference from explicit task-related strategies during natural language comprehension. Future studies may further examine how task demands influence such processes.

In conclusion, by combining the theoretically motivated HALM approach with sensitive multivariate neural analyses, this study provides clear electrophysiological evidence of the temporal dynamics of the labeling algorithm during natural language comprehension. Our results demonstrate that grammatical category labeling emerges around 200 ms—temporally coinciding with the operation of syntactic Merge—and dynamically continues into the N400 time window. This early labeling process serves as a real-time interface between the syntactic and conceptual–intentional systems, assigning grammatical roles and determining constituent heads to generate distinct phrase types. These findings reveal the parallel and interactive nature of syntax and semantics, thereby challenging strict syntax-first models and providing compelling neurobiological support for interactive accounts of language processing. Ultimately, this work advances our understanding of how the brain dynamically integrates formal and conceptual representations to generate hierarchical linguistic structures.

## ACKNOWLEDGMENTS

We thank Dr. Chen Zhao for her valuable suggestions on the syntax theory, and Xiaopu Hou for his help with the manuscript revision.

## FUNDING INFORMATION

Yiming Yang, Major Program of the Social Science Foundation of China, Award ID: 17ZDA301. Yiming Yang, National Natural Science Foundation of China (https://dx.doi.org/10.13039/501100001809), Award ID: 31271196. Yiming Yang, Major Entrusted Program of the National Social Science Foundation of China, Award ID: 20@ZH007.

## AUTHOR CONTRIBUTIONS

**Zhenghui Sun**: Conceptualization: Lead; Data curation: Lead; Formal analysis: Equal; Investigation: Lead; Methodology: Lead; Software: Equal; Visualization: Equal; Writing – original draft: Lead; Writing – review & editing: Lead. **Feipeng Chen**: Formal analysis: Equal; Visualization: Equal; Writing – review & editing: Supporting. **Shaodong Gui**: Data curation: Supporting; Methodology: Supporting; Writing – review & editing: Supporting. **Yajiao Shi**: Formal analysis: Equal; Writing – review & editing: Supporting. **Yiming Yang**: Funding acquisition: Lead; Project administration: Lead; Resources: Lead; Supervision: Lead; Validation: Lead; Writing – review & editing: Equal.

## DATA AND CODE AVAILABILITY STATEMENT

The data and analysis scripts related to the reported results are available on OSF: https://osf.io/9qvtx/?view_only=fd625500841b459d9f72433ce04ab0f7.

## Supplementary Material



## TECHNICAL TERMS

Labeling algorithm: The labeling algorithm assigns a grammatical category to a structure, determining its role within a phrase.
Merge: Merge is a basic operation that combines two elements into a hierarchical syntactic structure.
Parallel Interaction Model: The Parallel Interaction Model proposes that syntactic and semantic processes operate concurrently and dynamically interact during language comprehension.
Head-Anchored Labeling Manipulation (HALM): HALM is an experimental approach that varies phrase type by placing the same word in different structural positions.
Syntax-First Model: The syntax-first model proposes that syntactic structure is built before semantic interpretation begins.
