# Supplementary material for: The Temporal Dynamics of the Labeling Algorithm During Natural Language Comprehension: Neural Evidence for Phrase Grammatical Type Generation
Source: Neurobiol Lang (Camb). 2026 Jul 1;7:NOL.a.264. doi: 10.1162/NOL.a.264 (PMC13379301; doi:10.1162/NOL.a.264)
Supplement: Supplementary file 1 [file nol-07-264-s001.docx]

**The Temporal Dynamics of the Labeling Algorithm During Natural Language Comprehension: Neural Evidence for Phrase Grammatical Type Generation**

**Supplementary Information**
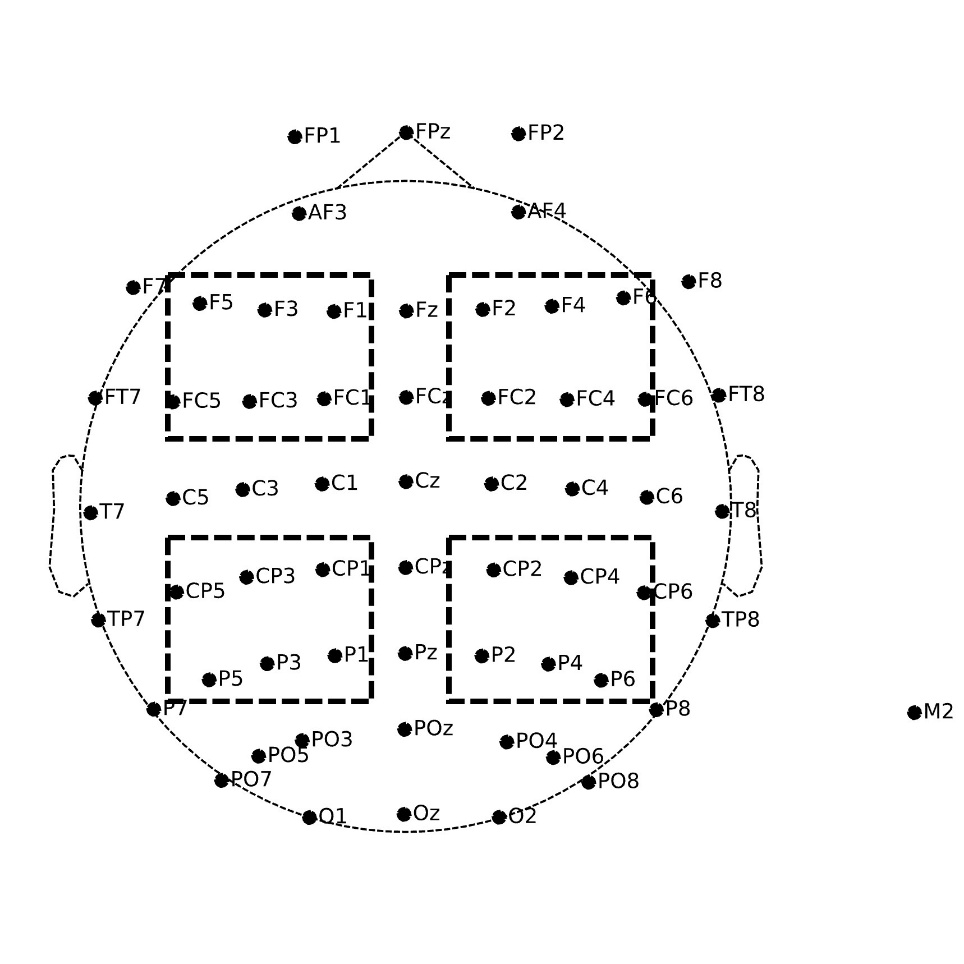


**Supplementary Figure 1:** Electrode locations and selection for the four regions of interest (ROIs). The electrodes are grouped as follows: left anterior (F1, F3, F5, FC1, FC3, FC5), right anterior (F2, F4, F6, FC2, FC4, FC6), left posterior (CP1, CP3, CP5, P1, P3, P5), and right posterior (CP2, CP4, CP6, P2, P4, P6). Each ROI is indicated by a dashed rectangular box.
